# Is childhood wheeze and asthma in Latin America associated with poor hygiene and infection? A systematic review

**DOI:** 10.1136/bmjresp-2017-000249

**Published:** 2018-02-22

**Authors:** Cristina Ardura-Garcia, Paul Garner, Philip J Cooper

**Affiliations:** 1 Clinical Sciences, Liverpool School of Tropical Medicine, Liverpool, UK; 2 Facultad de Ciencias Medicas, de la Salud y la Vida, Universidad Internacional del Ecuador, Quito, Ecuador; 3 Institute of Infection and Immunity, St George’s, University of London, London, UK

**Keywords:** childhood asthma, risk factors, infections, Latin America, hygiene hypothesis

## Abstract

**Introduction:**

High asthma prevalence in Latin-American cities is thought to be caused by poor hygiene and infections. This contradicts the widely accepted ‘hygiene hypothesis’ for asthma aetiology.

**Methods:**

Systematic review of observational studies evaluating the association between poor hygiene exposures or infections and asthma/wheeze among Latin-American children aged 4–16 years. MEDLINE, EMBASE, LILACS and CINAHL electronic databases were searched following a predefined strategy to 18 December 2017. We quantified outcomes measured and reported, assessed risk of bias and tabulated the results.

**Results:**

Forty-five studies included: 6 cohort, 30 cross-sectional and 9 case–control studies. 26 cross-sectional studies were school-based surveys (14 of over 3000 children), whereas 5 case–control studies were hospital/health centre-based. Exposures measured and reported varied substantially between studies, and current wheeze was the most common outcome reported. Data showed selective reporting based on statistical significance (P value <0.05): 17/45 studies did not clearly describe the number of exposures measured and 15/45 studies reported on less than 50% of the exposures measured. Most exposures studied did not show an association with wheeze or asthma, except for a generally increased risk associated with acute respiratory infections in early life. Contradictory associations were observed frequently between different studies.

**Conclusion:**

Selective reporting is common in observational studies exploring the association between environmental exposures and risk of wheeze/asthma. This, together with the use of different study outcomes (wheeze/asthma) associated with possibly distinct causal mechanisms, complicates inferences about the role of poor hygiene exposures and childhood infections in explaining asthma prevalence in Latin-American children.

## Introduction

Asthma prevalence has increased worldwide^1^ and is estimated to affect 400 million people.^2^ A widely accepted explanation for increased asthma prevalence in industrialised countries is provided by the ‘hygiene hypothesis’ in which diminished exposures to certain micro-organisms during the early years of life are purported to increase allergic disease risk.^3^

The immunological mechanisms underlying the hygiene hypothesis remain under debate. There is evidence that micro-organisms such as those present in the gut microbiota, nasal colonisers or intestinal helminths drive regulatory immune cells to maintain immune homeostasis. Consequently, reduced or altered exposures to such micro-organisms may lead to a failure in immune regulation, thus increasing the risk of chronic inflammation.^4^

Asthma is a heterogeneous disease caused by complex gene–environment interactions. It appears reasonable to attribute increasing asthma prevalence–occurring over a relative period of time–to changes in environmental exposures rather than in gene frequencies in human populations. Several studies have demonstrated a reduced risk of atopy and allergic diseases associated with farming,^5^ helminth infestations^6^ and contacts with other children. However, findings between studies are not always consistent,^7^ perhaps explained by the use of different asthma definitions, phenotypes (eg, atopic vs non-atopic) and diverse comparison groups.^8^ Inconsistent findings between studies may reflect also different underlying mechanisms and associated environmental exposures. Asthma is a complex disease likely consisting of several phenotypes, of which the most widely used are defined by the presence and absence of atopy. The proportion of asthma cases attributable to atopy is positively associated with economic development,^8^ and research in industrialised countries has mainly focused on atopic asthma, whereas non-atopic asthma, the predominant childhood asthma phenotype in Latin America,^8–10^ remains understudied.

Asthma prevalence may now be increasing in formerly low-risk, low-income and middle-income countries, while it has reached a plateau in many high-risk, high-income countries.^11^ High rates of asthma have been reported in some Latin-American cities^11^ in which conditions of overcrowding, poor hygiene and high infectious disease burdens predominate. Further, higher rates of asthma have been described in poorer Latin-American regions with a high prevalence of acute respiratory infections and intestinal parasite infestation in early childhood.^12^ Several Latin-American studies have shown that factors associated with poor hygiene may be associated with a higher risk of non-atopic asthma or wheezing.^9 13 14^ The role of chronic infections (eg, intestinal parasites) in the development of asthma in Latin America remains controversial: such exposures attenuate atopy but appear to have little impact on asthma prevalence.^15 16^

Our aim was to summarise and appraise the evidence of association between asthma or wheeze and (1) poor hygiene and (2) past and current parasite infections and chronic viral or bacterial infections.

## Methods

### Inclusion criteria

Studies were included if they met the following criteria (Annex 1): (1) observational study design (cross-sectional, cohort and case–control studies); (2) children aged 4–16 years, born and currently living in a Latin-American country; (3) asthma (guidelines criteria or reported doctor’s diagnosis), wheeze reported by written or video questionnaire (‘has your child/have you had wheezing during the last 12 months?’) or a doctor’s diagnosis, or bronchial hyper-responsiveness included as outcomes and (4) environmental exposures associated with a higher risk of infection or infections (gastrointestinal or respiratory infections, current/past intestinal parasites or chronic bacterial or viral infections) listed as exposures.

Exclusion criteria were: (1) reports not in English, Spanish, French or Portuguese; (2) published before 1980; (3) children outside Latin America involved as participants; (4) acute wheeze or asthma used as outcomes and (5) unpublished data and conference abstracts.

### Data sources and searches

We identified relevant studies by searching MEDLINE, EMBASE, LILACS and CINAHL electronic databases. No language, time or publication status restrictions were applied, and the last search was run on 28 December 2017 by CA-G together with an information specialist from Cochrane Infectious Diseases Group (online supplementary material: Annex 1). We used reference manager software (EndNote) to merge all the search results and remove duplicates.

10.1136/bmjresp-2017-000249.supp1 Supplementary data

Titles and abstracts were screened by CA-G to exclude studies. When abstracts were not available, tables and most relevant content were reviewed. Finally, full-text articles of selected papers were reviewed for eligibility as described above.

### Data extraction

We piloted our initial data extraction form with nine of the most relevant studies to develop the final form, which was used to retrieve data from selected studies. Data extraction was carried out by CA-G as prespecified in the study protocol and included: study characteristics (design, location, year and duration), participants (number, age range, sample selection and method of recruitment), outcomes (definitions and prevalence in study sample), exposures (type, methods used to assess exposure, time and duration of exposure and number of exposures measured) and results. Data were summarised in tables, and studies grouped by design. Where possible, results were presented and divided into atopic and non-atopic wheeze or asthma, with atopy defined as a positive skin prick test or positive specific serum IgE to any aeroallergen.

### Risk of bias assessment and data analysis

Potential risk of bias was described in relation to (1) sampling (random sampling and response rates); (2) reporting of the results (number of exposures described and reported to evaluate selective reporting) and (3) statistical analysis (statistical corrections for multiple significance testing). We reported if studies were adjusted for potential confounders and effect modifiers.

### Synthesis

We examined results by exposures related to the home environment, animal contact, contact with children, early-life infections and current/past infection with intestinal parasites. Results were reported together with the outcome used (wheeze or asthma).

## Results

**Figure 1:**
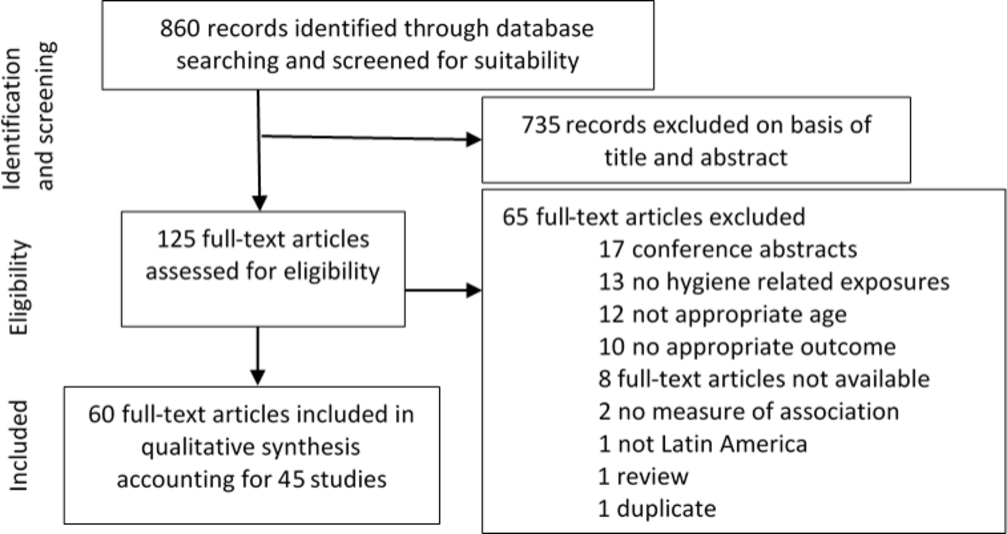
Flow diagram of included and excluded studies.

The search yielded a total of 860 reports of which 125 full-text articles were assessed for eligibility. Sixty reports representing 45 studies fulfilled the eligibility criteria (figure 1).

### Description of studies

#### Study design

The main characteristics of included studies are summarised in table 1. There were 6 cohort, 30 cross-sectional and 9 case–control studies. Twenty-seven (60%) studies were from Brazil, and the majority were urban-based, done between 1987 and 2014 (online supplementary table 1).

10.1136/bmjresp-2017-000249.supp2 Supplementary data

**Table 1:** Classification of exposures related to poor hygiene and infection

	Definition	Total (45)	Cohort (6)	Cross-sectional (30)	Case–control (9)
Home environment	Water and sanitation, garbage disposal, house cleaning, housing construction, endotoxins	9	2	6	1
Animal contact	Contact with pets, farm animals and cockroaches inside/outside the house	34	5	23	6
Contact with other children	Overcrowding in home, day-care attendance and having older siblings	22	6	11	5
Early-life infection	Acute respiratory and gastrointestinal infections during infancy, worm infections	11	4	6	1
Demonstrated infection	Intestinal parasites or chronic viral or bacterial infections diagnosed by serology or stool sample	21	4	11	6

Numbers represent the number of studies that measured at least one exposure related to each category, even if the results were not published.

Of the cross-sectional studies, 14 had a sample size of greater than 3000 subjects as recommended by the International Study of Asthma and Allergies in Childhood (ISAAC),^17^ whereas the rest ranged 100–2700 subjects. The majority were school-based.

As for the case–control studies, 6 were done in hospitals or health centres and 3 in schools or homes with samples varying: 400–600 children in 4 studies, 100–200 in 4 and 19 in 1.

#### Outcomes and case definition

The most frequent outcome was current or recent wheeze, as defined by the ISAAC question: ‘Has your child/have you had wheezing or whistling in the chest in the last 12 months?’.^18^ This was used in 5 cohort and 20 cross-sectional studies and was the case definition in 3 case–control studies. Some studies representing more than one report varied the outcome used (see footnote in online supplementary table 1).

Case definition for the remaining case–control studies was either doctor diagnosis of asthma or that defined by the Global Initiative for Asthma guidelines.^19^ Study outcomes used are provided in online supplementary table 1.

#### Measurement of exposures

Table 1 shows the exposures measured. Exposures relating to a higher risk of infection or specific infections varied greatly between studies. Although 5 of the cohorts, 20 of the cross-sectional and 4 of the case–control studies used ISAAC-derived questionnaires, the exposures measured differed greatly. Social Changes, Asthma and Allergy in Latin America (SCAALA) Brazil^9 15 16 20 21^ collected stool and blood samples to diagnose intestinal parasites and viral or bacterial infections. Two more cohort,^22–26^ 10 cross-sectional and 4 case–control studies analysed intestinal parasites (3 using serology). One other cohort^27^ and one cross-sectional study^28^ also collected blood to detect viral or bacterial infections.

Only two cohort^9 15 16 20–22 25^ and two cross-sectional^10 29–31^ studies measured at least one exposure variable in the five exposure categories. The most frequently measured exposure category was animal contact followed by contact with other children and infections (table 1).

### Risk of bias

#### Sampling

Sampling methods and response and follow-up rates are shown in online supplementary table 1. Ten of 30 cross-sectional studies used non-random (or unclear selection) samples. Eighteen of these 30 studies obtained a response rate ≥84%, 5 between 45% and 75% and 7 did not provide response rates.

#### Reporting of results

The risk factor questionnaire for the ISAAC Phase II study included 67 variables (10 hygiene or infection-related), whereas Phase III included 34 for 13–14 years (3 hygiene or infection-related) and 43 for 6–7 years (7 hygiene or infection-related).

One-third of papers (17/45) did not clearly describe number of exposures measured (table 2). Eleven (39%) of 28 studies that described measured variables reported on <50% of them, 3/28 (11%) reported on 50%–80% and 14/28 (50%) reported on more than 80% of the measured exposures. Among all studies: 15/45 (33%) reported on <50% of measured exposures, 8/45 (18%) on 50%–80%, 18/45 (44%) on >80% and 4/45 had no data.^32–35^

**Table 2:** Risk of bias: reporting of exposures measured, adjustment for confounders and proportion of variables associated with asthma or wheeze

Design	Study	ISAAC questionnaire	Exposures measured	Exposures reported	Criteria for presentation	Adjustment for confounders	Hygiene exposures with association (P<0.005)	Non-hygiene exposures with association (P<0.05)	Total exposures with association (P<0.005)
Cohort	Brandão *et al*^45 46^	Yes	43 (6–7 years)? 34 (13–14 years)?	26 21	Not described for univariate analysis. Variables reported varied between the different ages	Yes	3/7	3/15	6/22
	SCAALA Brazil^9 15 16 20 21^	Yes	55	50	‘Meaningfully associated’ in univariate or multivariate analysis (with *Schistosoma mansoni* and hookworm excluded from analysis)	Yes	4/28	1/22	5/50
	Pelotas cohort^23 24 26^	Modified	20?	20	Not described for multivariate. No hygiene exposure in multivariate*	Yes*	1/5	7/15	8/20
	Cuban study^60–62^	Modified	25	24	One more variable only used as confounder in multivariate	Yes	2/15	6/10	8/25
	ECUAVIDA^22 25^	Modified	47?	25	Not described, except confounders chosen for significant associations with the 5-year AW phenotype and on previously reported associations with asthma or with microbiome shifts	Yes	3/12	4/13	7/25
	Zepeda *et al*^27^	No	15	14	Not described	No	1/12	0/3	1/15
Cross-sectional	SCAALA Ecuador^10 30 31^	Yes	47	27	P<0.2 in univariate analysis or included in multivariate analysis. Parasites with low prevalence not represented	Yes	5/15	2/32	7/47
	Uruguaiana study^13 14^	Yes	41?	11–12	Risk factors known to be associated with wheeze/asthma and those significantly associated in the bivariate analysis. Intestinal parasites	Yes†	1/11	6/30	7/41?
	Azalim *et al*^32^	Modified	?	12	Not described	Yes	0/2	4/11	4/13
	Barraza *et al*^33^	Yes	?	16	Not described	Yes	2/3	8/13	10/16
	Barreto and Sole^28^	Yes	33	15	P<0.2 in univariate analysis and P<0.05 in bivariate analysis	No	3/?	12/?	15/33
	Bragagnoli and Silva^63^	Yes	18	15	Only presented results for the *Ascaris lumbricoides* coinfections with other parasites (not those other parasites alone)	No	3/14	2/4	5/18
	Casagrande *et al*^29^	Yes	33?	31	Not described (P<0.2 in univariate analysis to include in bivariate)	Yes	0/11	2/20	2/31
	Cooper *et al*^64^	Yes	4	3	Not specified (low proportion of *Ancylostoma duodenale*?)	Yes	0/3	–	0/3
	Del-Rio-Navarro *et al*^44^	Yes	43 (6–7 years) 34 (13–14 years)	6–10 7–14	Statistically significant results (for univariate and multivariate)	Unclear	1/1 8/13	7/9 1/1	8/10 9/14
	Endara *et al*‡ 65	Yes	1 (+6 parasites)	1	No association with intestinal parasites	Yes	0/1 (7)	0	0/1 (7)
	Freitas *et al*^66^	Yes	65	10	P<0.2 in univariate was included in multivariate. Final model variables P<0.05	Yes	1/10	2/55	3/65
	Garcia *et al*^67^	Yes	35	12	P<0.25 in bivariate analysis	Yes	1/3	5/32	6/35
	Gomes de Luna *et al*^68^	Modified	34	34		Yes	0/1	4/33	4/34
	Guimarães *et al*^69^	No	14	14		No	0/2	2/12	2/14
	Hagel *et al*^70^	–	2	2		No	0/2	–	0/2
	Han *et al*^71 72^	Modified	37?	21/26	Not specified	Yes§	2/13	11/24	13/37
	Kuschnir and Alves da Cunha^73^	Yes	34	10	Not stated	Yes	2/3	3/31	5/34
	Lima *et al*^74^	Modified	9	9		Yes	0/1	3/8	3/9
	Maia *et al*^75^	No	8	3	P<0.05 in final multivariate model	Yes	1/1	2/7	3/8
	Palvo *et al*^76^	Modified	14	9	P<0.2 in univariate analysis and P<0.05 in multivariate analysis	Yes	1/4	5/10	6/14
	Prietsch *et al*^77^	No	28?	17	P<0.2 in univariate analysis (to be included in multivariate)	Yes	1/6	6/22	7/28
	Quiroz-Arcentales *et al*^42^	No	15?	5	Not stated	No	4?/5	6?/10	10?/15
	Ribeiro *et al*^78^	No	17	7	Not stated	No	0/5	1/12	1/17
	Rojas Molina *et al*^34^	Modified	?	3–4	Only significant variables	No	1/?	2–3/?	3–4/?
	Silva *et al*^79^	No	1	1		Yes	0/1	-	0/1
	Solis-Soto *et al*^35^	Yes	?	9	Not specified	Yes	1/5	2/4	3/9
	Soto-Quiros *et al*^80^	No	10	10		No	1/1	4/9	5/10
	Souza *et al*^81^	–	1	0	No result for asthma alone (only for respiratory allergy)	No	0/1	–	0/1
	Tintori *et al*^82^	No	14?	14	P<0.5 in univariate was included in multivariate. Final model variables P<0.05	Yes	1/2	11/12	12/14
	Toledo *et al*^83^	Modified	12	4	Not stated	No	0/4	0/8	0/12
Case–control	Boneberger *et al*^43^	Modified	13	11	Matched by sex and age (not represented)	Yes	3/6	1/7	4/13
	Cadore *et al*^84^	No	14?	14		Yes	1/4	5/10	6/14
	Coelho *et al*^47^	Yes	67?	35	Not stated. P<0.2 in bivariate included in multivariate	Yes	2/11	4/24	6/35
	Jucá *et al*^85^	Yes	67?	26	Not stated. P<0.2 in bivariate included in multivariate	Yes	1/10	7/58	8/68
	López *et al*^86^	–	1	1		No	0/1	–	0/1
	Mendoza *et al*^87^	–	6	6		No	1/6	–	1/6
	Moraes *et al*^88^	No	22	22		Unclear¶	1/3	1/19	2/22
	Oliveira-Santos *et al*^89^	Yes	67?	26	Not stated. P<0.25 in bivariate included in multivariate	Yes	2/6	1/20	3/26
	Rizzo *et al*^90^	No	3	0	‘No significant difference’ in text, but no numbers represented	No	0/2	0/1	0/3

?: unclear number or not included.

*Muiño *et al*^26^: no description of variables measured and represented. No adjustment.

†Only for Pereira *et al*^14^ not da Silva *et al*.^13^

‡Same questionnaire as SCAALA Ecuador.

§Only rural residence and antibiotic use and bronchiolitis in the first year of life were adjusted for in multivariable analysis, as was the objective of the study.

¶Not published.

Exposures associated to wheeze or asthma: shown over total exposures measured or represented (when number of exposures measured was not clear). When results presented were divided in subgroups, highest number of associated variables from any of the subgroups was selected for the table.

AW, atopic wheeze; ISAAC, International Study on Asthma and Allergies in Children; SCAALA, Social Changes, Asthma and Allergy in Latin America.

The most common criteria used for presentation of results were statistical significance variables (P value <0.05 or <0.2) in univariate analyses which were then included in multivariate models (table 2).

#### Statistical analysis

None of the reports carried out any corrections for multiple significance testing.

### Measurement of confounders and effect modifiers

Thirty-six per cent (16/45) of papers did not show results adjusted for potential confounders, or information on adjustment was unclear (table 2).

Adjustment for several risk factors for asthma identified in previous published literature,^10 36–41^ which could act as confounders or effect modifiers are represented in online supplementary table 2. None of the studies were adjusted for all of the possible confounders (ie, age, gender, atopy, bronchiolitis in infancy, parental asthma, breastfeeding, socioeconomic status and tobacco exposure). Age, gender and parental asthma were most frequently adjusted for.

Atopy and history of bronchiolitis may behave as effect modifiers when studying risk factors for asthma.^10 38^ SCAALA Brazil,^9 15 16 20 21^ ECUAVIDA,^22 25^ SCAALA Ecuador^10 30 31^ and the Uruguaiana study^13 14^ represented results stratified by atopy, and another 13 studies adjusted for atopy. Only five studies adjusted for bronchiolitis. Some reports did not distinguish bronchiolitis from early-life respiratory infections.

### Results by environmental exposures

The high heterogeneity in methodology, exposures studied and outcomes measured precluded a meta-analysis. The main results are summarised in table 3. The results for the most relevant exposures are provided in online supplementary tables 3–7.

**Table 3:** Results for the association between exposures related to a higher risk of infection and wheeze/asthma

Study	Home environment						Animal contact					Contact with other children			Early-life infection		Demonstrated infection			
	House*	Sanitation†	Water‡	Endotoxin	Housing	Gar bage§	Inside	Outside	Pets	Farm animal	Insects¶	Overcrowding	Day care	Older sibling	ARI	GI	Intestinal parasite		Virus**	Bacteria**
																	Stool	IgG		
Brandão									NA			NA/↑^1^	NA		↑					
SCAALA Brazil	↑**^2^**	NA	NA			NA	↑**^2^**	NA	NA	–	NA	NA	↑**^2^**		↑**^2^**	NA	NA	NA^3^	NA	NA
Pelotas cohort							NA^4^		NA^4^			NA			↑**^5^**	NA**^5^**				
Cuban study									NA			NA	NA	NA			NA/↓ /↑^6^			
ECUAVIDA		NA	NA						NA	NA		NA		NA	↑	NA	↓/↑^7^			
Zepeda *et al*^27^													NA	NA					NA	
SCAALA Ecuador		↑^8^	NA/↑^9^		NA^9^		–	NA	NA/ ↑^10^	NA		NA	NA	NA^10^/↓^11^			NA/ ↓^12^			
Uruguaiana study							–	–	–	–		–	–	NA/↓^13^			NA/↓ /↑^14^			
Azalim *et al*^32^									NA			NA								
Barraza *et al*^33^							NA								↑					
Barreto and Sole^28^									↓										↑^15^	↑^15^
Bragagnoli and Silva^63^																	NA/↓ /↑^16^			
Casagrande *et al*^29^				NA				–	NA		–		NA	NA	NA		NA			
Cooper *et al*^64^																	NA			
Del-Ri­o-Navarro *et al*^44^									↑	↑										
Endara *et al*^65^																	NA			
Freitas *et al*^66^									↑			NA		↑			NA			
Garci­a *et al*^67^									↑**^17^**	–										
Gomes *et al*^68^									NA											
Guimarães *et al*^69^							NA					NA								
Hagel *et al*^70^																	NA			
Han *et al*^71^									NA	NA			NA	NA^18^	↑					
Kuschnir and Alves da Cunha^73^									↑					↓						
Lima *et al*^74^									NA											
Maia *et al*^75^									↑											
Palvo *et al*^76^									↑				–	–						
Prietsch *et al*^77^		NA	–		–				–			NA			↑					
Quiroz-Arcentales *et al*^42^		↑^19^	NA			↑	–	–							↑					
Ribeiro *et al*^78^					–				NA			–	NA	–						
Rojas *et al*^34^							↑			–				–						
Silva *et al*^79^																		NA		
Solis-Soto *et al*^35^		NA^20^				NA^20^			NA	NA	↑^21^									
Soto-Quiros *et al*^80^															↑					
Souza *et al*^81^																	NA			
Tintori *et al*^82^									NA/ ↑^22^											
Toledo *et al*^83^							NA	NA			NA									
Boneberger *et al*^43^									NA	↓			↓	NA	↑					
Cadore *et al*^84^									NA/ ↑^23^			NA						NA		
Coelho *et al*^47^							NA		NA			NA	NA/↑^24^	NA			NA			
Jucá *et al*^85^							↑	–				–	–	–						
López *et al*^86^																		NA		
Mendoza *et al*^87^																	↓			
Moraes *et al*^88^									↓		NA						NA			
Oliveira-Santos *et al*^89^									NA/ ↓^25^			NA		↓			NA^25^			
Rizzo *et al*^90^				NA																

1: increased risk of current wheeze at 6 years,^45^ no association at 13–14 years of age when adjusted^46^; 2: increased risk of non-atopic wheeze compared with non-atopic non-wheezing^9^: animals inside: rats; 3: only *Toxocara* IgG increased risk of atopic wheeze when compared with non-atopic non-wheezers^21^; 4: only in Chatkin *et al*^23^; 5: only in Muiño *et al*, 2008; 6: current helminth infection^62^ past *Ascaris lumbricoides*, *Trichuris trichiura* and hookworm infection (Werff 2013) were NA, history of *Ascaris* infection increased the risk of current wheeze^62^; 7: maternal geohelminths increased the risk of current wheeze, childhood geohelminths to 36 months decreased risk of current wheeze and asthma; 8: NA in Cooper *et al*^30^; 9: increased risk of wheeze with lack of potable drinking water in Cooper *et al*^30^; 10: dog inside the house increased risk of current wheeze in urban setting; 11: only in Cooper *et al*^30^; 12: no association for any geohelminth, hookworm or *Ascaris*^10 31^ ^30^, decreased risk of atopic wheeze with *T. trichiura* infection^10 31^; 13: decreased risk of active asthma, NA for wheeze; 14: high load of *Ascaris* increased risk of active asthma,^14^ *Giardia* infection decreased risk and high load of helminth infection increased risk^13^; 15: history of measles or tuberculosis increased the risk of asthma; 16: the risk of wheeze decreased with light *A. lumbricoides* infections and increased with heavy infections and with *A. lumbricoides* and *T. trichiura* coinfections; 17: no association in 13–14 year group; 18: bronchiolitis increased risk of current wheeze and asthma in 6–7 years; 19: absence of sewage disposal increased the risk of asthma; 20: precarious household conditions together as one exposure including: precarious floor, precarious walls, precarious source of water, precarious sewage system; 21: presence of disease vectors at home: fleas, ticks, kissing bugs, mice, bedbugs, flies; 22: dogs in the house increased the risk of current wheeze, no association for cats; 23: contact with cats increased risk of asthma, with dogs had no association; 24: kindergarten increased risk, day care was not associated; 25: dogs inside the house currently not associated, during 1 year of life decreased the risk of current wheeze. Worm infection in the past not associated with current wheeze.

*Infrequent house cleaning.

†No toilet or latrine.

‡No clean drinking water.

§No garbage disposal.

¶Cockroaches in the house.

**Positive serology for *Helicobacter pylori*, hepatitis A virus, herpes zoster virus, herpes simplex virus, Epstein-Barr virus.

ARI, acute respiratory tract infection; GI, gastrointestinal;; SCAALA, Social Changes, Asthma and Allergy in Latin America.

–: exposure measured not represented; NA: no association (P≤0.05); ↑: increased risk of asthma (OR>1, P<0.05); ↓: decreased risk of asthma (OR<1, P<0.05).

#### Home environment

Water and sanitation infrastructure, garbage disposal, frequency of house cleaning, housing construction materials and presence of endotoxins were analysed in nine studies (table 1). Most results showed no association, except for an increased risk of current wheeze with open-field defecation compared with toilets or latrines (adjusted OR (AOR) 1.31, 95% CI 1.02 to 1.68)^10^ and with lack of potable drinking water (AOR 1.44, 95% CI 1.16 to 1.78),^30^ an increased risk of asthma with the presence of sewage disposal^42^ and increased risk of non-atopic wheeze compared with non-atopic non-wheezing with infrequent house cleaning.^9^

#### Animal contact

The association between animal contact and wheeze was investigated in 34 studies (table 1), though only reported in 30 studies (online supplementary table 3). All studied the effect of domestic animals (11 with cats and 9 with dogs), with inconsistent findings. Farm animals were evaluated in 10 studies, one showed a decreased risk of asthma after regular contact,^43^ another showed an increased risk of cumulative asthma in boys aged 6–7 years after maternal contact with animals during pregnancy,^44^ with no associations (or no results presented) in 8 other studies. Overall, there was no consistent effect that might reflect protection or causality.

#### Contact with other children (older siblings, overcrowding and day-care attendance)

Of 12 studies reporting the relationship with having older siblings, there was no obvious pattern (online supplementary table 4). The 13 studies evaluating the effect of overcrowding did not report on an association, except for Brandão cohort,^45^ which showed an increased risk of current wheeze at 6 years among children born in private hospitals and not exposed to household overcrowding (AOR 6.46, 95% CI 1.11 to 37.57)^45^ and no association at 13–14 years.^46^ Day-care attendance increased the risk of non-atopic wheeze in a cohort study^9^ and of asthma in a case–control study,^47^ decreased the risk of asthma in another case–control study^43^ and showed no association with wheeze or asthma in eight other studies (online supplementary table 5).

#### Early-life infections

Online supplementary table 6 shows the effects of early-life infections on wheeze and asthma. Nine studies demonstrated an increased risk of wheeze and asthma (both atopic and non-atopic) associated with acute respiratory infections in early life, though the cohort study that reported AOR found an association with non-atopic wheeze and not with atopic wheeze.^9^ A further cross-sectional study found no association between viral bronchiolitis and recurrent wheezing.^27^ Gastrointestinal infections in early life showed no association with wheeze or asthma in three studies.^9 22 26^

#### Intestinal parasites

Intestinal parasites were analysed in 18 studies (online supplementary table 7), reporting no association with wheeze or asthma in 11. Positive and negative associations reported in the remaining seven studies varied greatly depending on the specific parasite, load of infestation, presence of coinfections and age of exposure (intrauterus, early life or current).

## Discussion

Overall, current evidence is not sufficient to derive a conclusion as to whether poor hygiene exposures and early-life infections affect the risk of developing childhood wheeze or asthma in Latin America. Only six cohort studies were included in this review, five of which followed up children from the first few years of life, though only one was specifically designed to study asthma outcomes. Selective reporting of statistically significant results was common to many studies (with the exception of the cohort studies), exposure variables measured varied greatly between studies and the majority of studies showed no associations with asthma or wheeze. The exception was early-life acute respiratory infections, which showed reasonably consistent positive associations with wheeze (mainly non-atopic) and asthma across studies.

The use of a wide literature search with no language restriction and including a Latin-American database probably identified the majority of relevant studies. The inclusion of studies from several South and Central American countries ensured the representation of different Latin-American regions. Most cross-sectional studies included in this review were methodologically of good quality following the ISAAC guidelines^17 18^ and included large sample sizes. The use of a widely validated questionnaire such as the ISAAC questionnaire^18 48 49^ in a large proportion of studies provided a reasonably standardised instrument to measure exposures and wheeze or asthma.

Substantial selective reporting was observed across studies, with a large or even unknown number of exposures studied and only statistically significant variables reported. Similarly, none of the studies applied any statistical correction for multiple testing, even though more than 30 variables were studied in some reports, increasing the risk of type I statistical errors. Selective reporting, together with a large number of tested associations, small effect sizes, differences in design, definitions, outcomes and analytical approaches used, may produce spurious associations.^50^ This may have biased the overall understanding of the role of environmental exposures on the development of asthma or wheeze in Latin-American children. A part of the observed selective reporting may be explained by publication bias, reflecting difficulties in publishing negative or non-conclusive findings and leading to selective reporting of positive results. However, recent provisions for online supplementary tables for most publishing platforms now allow authors to provide data and associations for all exposures measured.

Early-life infections have been shown to protect against atopy,^15^ but effects on asthma are still controversial. Evidence in this review points towards a higher risk of wheeze or asthma associated with early-life respiratory infections. Only five studies collected this information prospectively,^9 25–27 45 46^ and four of which reported an increased risk of wheeze or asthma following early-life respiratory infections.^9 25 26 45 46^ Respiratory syncytial virus bronchiolitis is considered to be an important risk factor for asthma,^51^ whereas rhinovirus has been associated with acute asthma exacerbations.^52 53^ These findings are difficult to interpret as most studies do not describe the type of respiratory infection or whether such infections were simply a manifestation of their underlying respiratory disease (transient wheeze or asthma). On the other hand, gastrointestinal and other chronic viral or bacterial diseases may not affect the risk of wheeze or asthma in Latin America.^15^

The association between intestinal parasites (mainly geohelminths) and asthma has been widely studied, and although a protective effect on atopy has been demonstrated,^6 10 15^ their effects on asthma remain unclear. An international meta-analysis showed no overall effect on asthma, though *Ascaris lumbricoides* was associated with an increased risk and hookworm with a decreased risk.^54^ Similar findings can be seen in this review, with a predominantly protective effect of *Trichuris trichiura* on atopic wheeze and a higher risk of asthma or wheeze associated with *A. lumbricoides* infestation. The effect of intestinal helminths on asthma may depend on many factors, such as parasite species, intensity of infection, age of first infection and duration of infection.^55^

Animals living around the home may increase the risk of infection with certain pathogens associated with asthma (eg, *Toxocara canis*).^55^ Here, pet contact was not clearly associated with a higher risk of wheeze/asthma. A meta-analysis of international studies^56^ found that dog exposures increased the risk of asthma slightly, whereas cat exposures reduced the risk. As furry animals may induce allergic diseases, it is difficult to ascertain whether they may increase the risk of asthma by increasing the risk of early-life infections or through their effect on atopy. Consistent protective effects across studies of contact with farm animals against asthma are one of the most compelling observations in support of the hygiene hypothesis.^5^ This review provides only limited support for a protective role of such exposures in Latin-American populations.

Overcrowding, day-care attendance and having older siblings may increase the risk of early-life infections due to frequent and close contact with other children. However, there is no clear evidence of the effect these exposures have on childhood asthma.^57^ In this review of Latin-American studies, these exposures in general were not associated with wheeze or asthma.

This review has several limitations. First, most of the studies included in the review were cross-sectional or case–control studies, which preclude establishing a time association between exposures and outcome. Second, the definition of asthma or wheeze differed between studies, complicating the analysis as not all wheeze is asthma, and although current wheeze is a good indicator of asthma for prevalence studies,^2^ it may not be suitable for exploring asthma risk factors. More importantly the symptom ‘wheeze’ may be a manifestation of other respiratory pathologies, such as childhood respiratory infections that are a more frequent cause of chronic respiratory symptoms in Latin America than in other regions.^12^ Within ‘wheeze’ may be included different disease processes with differing risk factors, as indicated by the observation from a recent meta-analysis of observational studies from industrialised countries that endotoxin exposure may increase the risk of wheeze in younger children but be protective against asthma in older children.^58^ Asthma/wheeze likely encompasses a range of phenotypes and wide spectrum of disease severity associated with different patterns of risk factors. However, with the available data in this systematic review, it was not our aim to evaluate the effects of poor hygiene and infections on disease phenotypes or severity. Third, two-thirds of the studies were done in Brazil, with scarce representation of other large urban centres such as those present in Argentina, Peru or Uruguay. This may limit the generalisability of the findings to other Latin-American countries with different circumstances such as climate, socioeconomic level or diet. Finally, most studies did not provide results stratified by atopy, an important effect modifier. Previous studies have found contradictory effects of certain factors related to microbial exposure on either atopic or non-atopic asthma.^59^

## Conclusion

In conclusion, our findings in this systematic review do not settle the debate of whether the hygiene hypothesis is relevant or not to the high asthma prevalence in Latin-American children. Our analysis indicates a higher risk of wheeze and asthma in Latin America associated with acute respiratory infections in early life. Highly heterogeneous results regarding poor hygiene and early-life infections may be explained by difference in asthma phenotypes (atopic vs non-atopic) and control groups used for comparison as well as different definitions used (current wheeze vs doctor’s diagnosis of asthma). Selective reporting is common among observational studies exploring associations between environmental exposures and wheeze or asthma risk. Large prospective cohort studies with standardised outcomes are needed in Latin America to clarify the role of poor hygiene exposures and early-life infections on the development of childhood wheeze and asthma. Such studies should help guide policy makers on decisions of potential strategies to reduce the high asthma burden in Latin America.
