# Supplementary material for: Is childhood wheeze and asthma in Latin America associated with poor hygiene and infection? A systematic review
Source: BMJ Open Respir Res. 2018 Feb 22;5(1):e000249. doi: 10.1136/bmjresp-2017-000249 (PMC5844372; doi:10.1136/bmjresp-2017-000249)
Supplement: Supplementary data [file bmjresp-2017-000249supp001.pdf]

Search report Risk factors for asthma in Latin America

Search No.	Date	Database searched	Search terms used	Hits (before duplicate removal)
1	18/12/2017	Medline (Pubmed)	See search terms below	222
2	18/12/2017	EMBASE (OVID)	See search terms below	583
3	18/12/2017	LILACS (BIREME)	See search terms below	268
4	18/12/2017	CINAHL (EBSCO)	See search terms below	28
**FINAL NUMBER OF REFERENCES IN ENDNOTE AFTER DELETING DUPLICATES =860**				

Pubmed Search terms

1	child OR children OR childhood OR infant* OR schoolchild* OR pediatr* OR paediatr* Field: Title/Abstract
2	“factor*” OR “dirt” OR “infection*” OR “hygiene*” OR “sanitation” OR “farm” OR “farming” OR “worms” OR “parasite*” OR “helminth*” OR “protozo*” OR “virus” OR “viral” OR “endotoxin*” OR “bacteria*” OR “drink* water” Field: Title/Abstract
3	#1 and #2
4	"Asthma"[Mesh]
5	asthma OR wheez* OR "bronchial hyper*" Field: Title/Abstract
6	#4 or #5
7	#3 and #6
8	""Latin America" OR Brazil OR Venezuela OR Colombia OR Argentina OR Chile OR Bolivia OR

	Argentina OR Ecuador OR Guyana OR Paraguay OR Suriname OR Uruguay Field: Title/Abstract
9	((("Caribbean Region"[Mesh]) OR "Central America"[Mesh]) OR "Gulf of Mexico"[Mesh]) OR "Latin America"[Mesh]
10	Belize OR "Costa Rica" OR " El Salvador" OR Guatemala OR Honduras OR Nicaragua OR Panama OR Cuba. Field: Title/Abstract
11	#8 OR #9 OR #10
12	#7 and #11

EMBASE Search terms

1) (child or children or childhood or infant* or pediatr* or paediatr*).mp. [mp=title, abstract, subject headings, heading word, drug trade name, original title, device manufacturer, drug manufacturer, device trade name, keyword]
2) (risk factor* or dirt or infection* or hygiene* or sanitation or farm or farming or worms or parasit* or helminth* or protozo* or virus or viral or endotoxin* or bacteria* or drink* water).mp.
3) 1 and 2
4) (asthma or wheezing or bronchial hyper*).mp.
5) 3 and 4
6) (Latin America or Brazil or Venezuela or Colombia or Argentina or Chile or Bolivia or Argentina or Ecuador or Guyana or Paraguay or Suriname or Uruguay).mp.
7) (Belize or Costa Rica or El Salvador or Guatemala or Honduras or Nicaragua or Panama or Cuba).mp.
8) (Caribbean Region or Central America or Gulf of Mexico or Latin America).mp.
9) 6 or 7 or 8
10) 5 and 9

LILACS search terms

(asthma OR wheezing) AND (child$ OR infant$ OR pediatr$ OR paediatr$) [Words] and (risk OR dirt OR hygiene OR sanitation OR farm$ OR worms OR parasite$ OR helminth$ OR viral OR virus OR environment$) [Words] and Brazil or Venezuela or Colombia or Argentina or Chile or Bolivia or Argentina or Ecuador or Guyana or Paraguay or Suriname or Uruguay OR Belize or Costa Rica or El Salvador or Guatemala or Honduras or Nicaragua or Panama or Cuba [Words]

CINAHL Search terms

1) AB (Brazil or Venezuela or Colombia or Argentina or Chile or Bolivia or Argentina or Ecuador or Guyana or Paraguay or Suriname or Uruguay OR Belize or Costa Rica or El Salvador or Guatemala or Honduras or Nicaragua or Panama or Cuba) OR TI (Brazil or Venezuela or Colombia or Argentina or Chile or Bolivia or Argentina or Ecuador or Guyana or Paraguay or Suriname or Uruguay OR Belize or Costa Rica or El Salvador or Guatemala or Honduras or Nicaragua or Panama or Cuba)

2) AB (asthma OR wheezing) AND AB (child OR children OR childhood OR infant* OR pediatr* OR paediatr*) AND AB (Search “risk factor*” OR “dirt” OR “infection*” OR “hygiene*” OR “sanitation” OR “farm” OR “farming” OR “worms” OR “parasite*” OR “helminth*” OR “protozo*” OR “virus” OR “viral” OR “endotoxin*” OR “bacteria*” OR “drink* water”)

3) 1 and 2
