# Supplementary material for: Is childhood wheeze and asthma in Latin America associated with poor hygiene and infection? A systematic review
Source: BMJ Open Respir Res. 2018 Feb 22;5(1):e000249. doi: 10.1136/bmjresp-2017-000249 (PMC5844372; doi:10.1136/bmjresp-2017-000249)
Supplement: Supplementary data [file bmjresp-2017-000249supp002.pdf]

Supplementary Table 1: Studies' Characteristics

Study Design	Study	Location	Urban / rural	Sample selection and setting	Age (years)	Sample size	Years	Outcomes measured
**Cohort**	Brandao [32, 33]	Brazil (Feira de Santana)	Urban	Prospective birth cohort with nested cross-sectional study at 6 years old. LTFU: 1.8%	6	672	2003-2009	Current wheeze
	SCAALA Brazil[9, 15, 16, 20, 21]	Brazil (Salvador)	Urban	Cross-sectional study derived from a previous cohort with prospective data on a few infectious exposures. Random selection of children from 24 sentinel areas without sanitation. Household survey LTFU rate: 9.4% (complete data: 80-91%)	4-11	1445	1997-2005	Current wheeze and asthma symptoms
	Pelotas Cohort [23, 24, 26]	Brazil (Pelotas)	Urban	Prospective birth cohort from 5 city hospitals. Nested cross-sectional studies at 4-5 and 6-7 years old of a 20% and 10% random sample, respectively. Follow-up: 11 years. LTFU rate: 12.5%	Birth to 10-12	981	1993-2004	Current wheeze and asthma
	Cuban Study[34-36]	Cuba (San Juan y Mart. and Fomento)	Urban and rural	Stratified random sample of schools. School-based survey. RR 100% Non-asthmatic children (1042) were followed-up 3 years after. LTFU: 23%	4-14	1321 (801 cohort)	2003-2007	Current wheeze and asthma (Doctor's dx)
	ECUAVIDA[22, 25]	Ecuador (Esmeraldas)	Rural	Birth cohort and nested case-control study at 5 years of age. LTFU rate: 10.6%	Birth to 5	2090 (97 case-control)	2005-2009	Current wheeze and asthma
	Zepeda 2016[27]	Chile (Santiago de Chile)	Urban	Prospective cohort study of infants >1 month old hospitalized for bronchiolitis. Follow-up until 4-5 years old.	4-5	22	2009-2014	Recurrent wheezing
**Cross-sectional**	SCAALA Ecuador[10, 30, 31]	Ecuador (Esmeraldas)	Urban and rural	Cross-sectional and nested case-control study. Convenience sample of 58 small communities (<250 pupils/school) and 11 urban schools. School-based survey. RR: 95%	6-16	6821	2005-2010	Current wheeze
	Uruguaia-na Study [13, 14]	Brazil (Uruguaiana)	Urban (poor)	Cross-sectional and nested case-control study. Simple random (not specified). School-based survey RR: 96%	9-13	1982	?	Current wheeze, active asthma, BHR
	Azalim 2014[37]	Brazil (Belo Horizonte)	Urban	Random sample of schools. School –based survey RR: 87%	6-7 & 13-14	706 596	?	Current wheeze
	Barraza 2003[38]	Mexico (Ciudad Juarez)	Urban	Random sample of schools. School –based survey RR: 92%	6-8 & 11-14	6200	?	Current wheeze
	Barreto 2014[28]	Brazil (Belem)	Urban	Random (by convenience) sample of schools. School –based survey. RR: ?	13-14	3725	2008-2009	Current wheeze
	Bragagnoli 2014[39]	Brazil (Campica Grande)	Urban (slum)	Home visits in a slum neighbourhood (all). RR: 98% (Faecal samples: 75%)	2-10	1004	2007	Current wheeze (≥episodes last 12m)
	Casagrande 2008[29]	Brazil (Sao Paulo)	Urban	Random sample of schools. School-based survey RR: 68%	6-7	561	2002	Current wheeze
	Cooper 2003[40]	Ecuador (Pichin. + Esmer.)	Rural	Convenience sample of schools RR: 96% quest, 88% stools	5-18	4601	?	Current wheeze
	Del Río 2006[41]	Mexico (Mexico City)	Urban	Random sample of schools. School-based survey RR: >90%	6-7 & 13-14	3000 3000	2002-2003	Current wheeze
	Endara 2010**[42]	Ecuador (Esmeraldas)	Rural	Convenience samples of schools receiving anthelminthic intervention and non-treated schools. School-based survey. RR: ?	6-16	3901	2005-2007	Current wheeze
	Freitas 2012[43]	Brazil (Outeiro and Combú islands)	Urban and rural (50%)	No information on randomization. School –based survey RR: near 100%	5-8	400	2007-2009	Current wheeze
	García 2008[44]	Colombia (Bogotá)	Urban	Random sample of schools (only SES 3-4). School survey RR: 90/99%	6-7 & 13-14	3256 3829	2002	Current wheeze
	Gomes L. 2015[45]	Brazil (Fortaleza)	Urban	Random sample of schools. School-based survey RR: 98.9%	13-14	694 (3015)*	2006-2007	Current asthma (questionnaire)

	Guimaraes 2015[46]	Brazil (Jequié)	Urban	4 public schools in convenience quarter (largest school in the city). School-based survey. RR: 60.9%	6-7	280	2011	Asthmatic symptoms (ISAAC questionnaire)
	Hagel 2007[47]	Venezuela (Caracas, El Cardón, La Salina)	Urban slum and rural	Non-random. School-based survey RR: ?	6-12	470	?	BHR
	Han 2017 [48, 49]	Argentina (San Francisco)	Urban and rural	School-based survey (all schools in the area). RR: 85.9% for 6-7 year olds, 99.8% for 13-14 year olds.	6-7 & 13-14	1517 1804	2007	Current wheeze and current asthma
	Kuschnir 2007[50]	Brazil (Rio de Janeiro)	Urban and semi-rural	Random sample of schools. School-based survey RR: ?	13-14	3033	2002	Asthma (question.)
	Lima 2012[51]	Brazil (Sao Luis)	Urban	Random sample of schools followed by random sample of students. School-based survey. RR: 89%	13-14	3069	2008-2009	Current wheeze
	Maia 2004[52]	Brazil (Montes Claros)	Urban and rural (9%)	Stratified random sample of schools followed by random sample of classes. School-based survey. RR: 97%	13-14	3770	2000	Current wheeze
	Palvo 2008[53]	Brazil (Sao Jose do Rio Preto)	Urban	Random sample of schools. School-based survey RR: 45% of schools 64% of children	6-7	2444	2003-2004	Current wheeze
	Prietsch 2006[54]	Brazil (Cidade de Rio Grande)	Urban	Cluster random (city sectors). Household survey RR: 88% found	7-12	685	2004	Recurrent wheeze
	Quiroz 2013[55]	Colombia (Coal regions)	Urban and rural	Stratified random sample. Household survey	<10	1627	?	Asthma (ISAAC)
	Ribeiro 2002[56]	Brazil (Sao Paulo)	Urban	Non-random sample. School-based survey RR: 72%	4-9	183	1996	Active asthma
	Rojas 2001[57]	Mexico (Guerrero and Acapulco)	Urban and rural	Non-random sample. School-based survey RR: ?	6-7 & 13-14	3754 3510	1999-2000	Current wheeze
	Silva 2016[58]	Brazil (Salvador)	Urban	Schools with ≥ 150 students (8/22 schools). School-based survey. RR: 66.6% (complete data)	6-13	791	2010	Wheezing/asthma? (Questionnaire)
	Solis-Soto 2013[59]	Bolivia (Oropeza)	Urban and rural	Random sample of schools. School-based survey. RR: 84% of schools, 91% of students	9-15	2340	2011	Current wheeze
	Soto 1994[60]	Costa Rica	Whole country	Stratified random sample of schools. School-based survey. RR: 89%	5-17	2682	1987	Asthma (Doctor's dx.)
	Souza 2012[61]	Brazil (Recife)	Urban	Non-random sample of patients from several health centres. RR: ?	5-15	110	2007	Asthma (quest.)
	Tintori 2013[62]	Brazil (Maringa)	Urban	Stratified random sample of schools. School-based survey. RR: 97%	12-15	3057	?	Current wheeze
	Toledo 2011[63]	Brazil (Taubaté)	Urban	Random sample of schools. School-based survey RR: 91%	13-14	809	?	Current wheeze
**Case-Control**	Boneberger 2011[64]	Chile (Valdivia)	Urban (>90%)	Hospital-based study. Controls: patient from hospital /primary care, no dx asthma, matched age + sex	6-15	482 (188 cases)	2008-2009	Asthma (GINA)
	Cadore 2016[65]	Brazil (Rio Grande)	Urban	Hospital-based study. Controls: children without asthma attending outpatient clinic for routine health care.	6m-12 years	208 (156 cases)	2012-2013	Asthma (GINA)
	Coelho 2016[66]	Brazil (Montes Claros)	Urban	Family Health Strategy program. (Clinic or home) Case-control nested in cross-sectional study of 1131 children. All asthmatics for cases, simple random for controls. RR: 75% cases, 82% controls.	6-14	551 (172 cases)	2007-2008	Current wheeze
	Juca 2012[67]	Brazil (Cuibá)	Urban	School-based study. Controls: Unclear (Not asthmatics)	13-14	590 (198 cases)	2008	Current wheeze
	Lopez 2009[68]	Argentina (Ciudad de Resistencia)	Urban?	Hospital-based study. Controls: No signs/symptoms asthma and similar age, SES, residency.	3-13	100 (47 cases)	?	Asthma (Doctor's dx)
	Mendoza 2008[69]	Colombia (Santa Marta)	Urban	Health centre based study. Controls: No asthma and no personal/family Hs of allergic diseases.	6-16	227 (97 cases)	?	Asthma (GINA)
	Moraes 2001[70]	Brazil (Mato Grosso)	?	Hospital-based study. Controls: No wheeze and no personal Hs of allergic diseases.	4-14	163 (59 cases)	1999	3 wheezing attacks last 2y
	Oliveira-Santos 2014[71]	Brazil (Aracaju)	Urban	Case-control nested in school-based survey of random selection of students from 70 schools proportionally distributed. Random sample of 35 schools for case-control study. Controls: non-asthmatic	13-14	430	2011-2012	Current wheeze

	Rizzo 1997[72]	Brazil (Sao Paulo)	Urban	Hospital-based study. Controls: Paediatric Emergency Room, no Hs allergic diseases, negative SPT.	6-16	19 (9 cases)	1993-1994	Atopic asthma (Doctor's dx.)

?: no information provided or unclear; BHR: bronchial hyperresponsiveness; dx: diagnosis; GINA: diagnosis of asthma using GINA (Global Initiative for Asthma) guidelines; Hs: history; LTFU: lost to follow-up; quest: diagnosis of asthma through a questionnaire; RR: response rate; SCAALA: Social Changes, Asthma and Allergy in Latin America; SES: socioeconomic stratum; SPT: Skin Prick Test; y: years. *: Only used 694 for the analysis (those who answered yes (current asthma) or no (no asthma) to both "wheezing in the past 12 months'' and ''asthma ever''). **: Asthma defined as parentally-reported wheeze during the previous 12 months plus one or both of parentally reported wheeze up to 3 years and a doctor diagnosis of asthma.

SCAALA-Brazil did not use the same outcome in all 4 reports, initially analysing subjects as atopic and non-atopic wheezers, and then later defining asthma (atopic and non-atopic) as current wheeze together with at least 1 of the following: i) diagnosis of asthma ever, (ii) wheezing with exercise in the last 12 months, (iii) ≥4 episodes of wheezing in the last 12 months or (iv) waking up at night because of wheezing in the last 12 months. SCAALA Ecuador presented their results for current wheeze, as well as separately for atopic and non-atopic wheeze. In the Pelotas Study, both 'current wheeze' and 'current asthma' (doctor diagnosis of asthma in the last 12 months) were used in both cross-sectional studies, while a classification of transient (up to 4 years old), persistent (on-going at 11 years of age) or late-onset wheeze (newly appeared at 11 years of age) was used for the cohort analysis. The cross-sectional component of the Uruguaiana study used current wheeze and active asthma (wheeze last 12 months plus asthma ever), giving separate results for non-atopic wheeze and active asthma, while current wheeze and bronchial hyper-responsiveness were evaluated in the case-control analysis.

Supplementary Table 2: Confounders and effect modifiers for the association between exposures related to higher risk of infection and wheeze/asthma.

Study	Age	Gender	Atopy	Bronchiolitis	Parental asthma	SES	Parental education	Tobacco exposure	Breastfeeding	Humid household
**Brandao**	Yes	Yes	No	No	Yes	Yes	No	Yes	No	No
**SCAALA Brazil**	Yes	Yes	Yes	No	Yes	No	Yes	Yes[1]	No	Yes[1,2]
**Pelotas Study**	Yes	Yes[3]	Yes[3]	Yes[3]	Yes[3]	Yes[3]	Yes[3]	Yes[3]	Yes[3]	No
**Cuban Study**	Yes	Yes	Yes[4]	No	Yes[5]	Yes[5]	Yes[6]	Yes[6]	No	No
**ECUAVIDA[7]**	Yes	Yes	Yes	No	Yes	Yes	Yes	No	No	No
**Zepeda 2016**	No	No	No	No	No	No	No	No	No	No
**SCAALA Ecuad.**	Yes	Yes	Yes	No	Yes	Yes	Yes	Yes[8]	Yes[9]	No
**Urug. Study[10]**	Yes	Yes	Yes	Yes	Yes	No	Yes	Yes	No	Yes
**Azalim 2014**	No	Yes[11]	No	No	No	No	Yes[11]	Yes	No	Yes
**Barraza 2003**	Yes	Yes	No	No	Yes	Yes[1]	Yes[1]	Yes	Yes	Yes
**Barreto 2013**	Yes	No	No	No	No	No	No	Yes	Yes	Yes
**Bragagn. 2014**	No	No	No	No	No	No	No	No	No	No
**Casagran 2008**	Yes	Yes	Yes	No	Yes	No	No	Yes	Yes	Yes
**Cooper 2003**	Yes	Yes	Yes	No	No	No	No	No	No	No
**Del Río 2006**	Yes	Yes	No	No	No	No	No	No	No	No
**Endara 2010[12]**	Yes	Yes	No	No	No	Yes	Yes	No	No	No
**Freitas 2012**	No	No	Yes[1]	No	Yes	No	No	No	No	Yes[1]
**García 2008**	Yes	No	No	No	No	No	Yes	Yes	No	No
**Gomes L 2015**	No	No	No	No	No	Yes	Yes	No	No	No
**Guimar. 2015**	No	No	No	No	No	No	No	No	No	No
**Hagel 2007**	No	No	No	No	No	No	No	No	No	No
**Han 2017**	Yes	Yes	No	Yes	Yes	No	No	Yes[13]	No	No
**Kuschnir 2007**	Yes	Yes	Yes	No	No	No	No	Yes	No	No
**Lima 2012**	Yes	No	Yes	Yes	Yes	No	No	Yes	No	No
**Maia 2004[14]**	Yes	No	No	No	Yes	No	No	No	No	No
**Palvo 2008**	Yes	No	Yes	No	Yes	No	No	Yes	No	No
**Prietsch 2006**	No	No	Yes	No	Yes	No	Yes	No	Yes	No
**Quiroz 2013**	No	No	No	No	No	No	No	No	No	No
**Ribeiro 2002**	No	No	No	No	No	No	No	No	No	No
**Rojas 2001**	Yes	No	No	No	No	No	No	No	No	No
**Silva 2016**	Yes	Yes	No	No	No	Yes	Yes	No	No	No
**Solis-Soto 2013**	Yes	Yes	No	No	No	No	No	Yes	No	No
**Soto 1994**	No	No	No	No	No	No	No	No	No	No
**Souza 2012**	No	No	No	No	No	No	No	No	No	No
**Tintori 2013**	No	No	No	No	Yes	No	No	Yes	No	Yes
**Toledo 2011**	Yes	No	No	No	No	No	No	No	No	No
**Boneb. 2011**	Yes	Yes	Yes	No	Yes	No	No	Yes	Yes	Yes
**Cadore 2017**	Yes	Yes	No	No	Yes	Yes	Yes	No	No	No
**Coelho 2016**	No	No	Yes	No	Yes	No	No	Yes	No	No
**Juca 2012**	Yes	Yes	No	No	Yes	Yes	No	Yes	Yes	Yes
**Lopez 2009**	Yes	No	No	No	No	Yes	No	No	No	No
**Mendoza 2008**	No	No	No	No	No	No	No	No	No	No
**Moraes 2001**	Yes	Yes	Yes	No	Yes	No	No	No	No	No
**Oliveira 2014**	Yes	No	Yes	No	Yes	No	No	Yes	Yes	Yes
**Rizzo 1997**	No	No	No	No	No	No	No	No	No	No

SCAALA: Social Changes, Asthma and Allergy in Latin America; 1: Potential confounders discarded from final multivariate logistic regression model if not statistically significant ($p<0.05$) by using a stepwise process; 2: Included in final model in Mendonça 2012, when analysing Toxocara IgG seropositivity as exposure; 3: Not in the cohort study up to 11 years of age (Muiño 2008); 4: Only in Kanobana 2013 and Werff 2013; 5: Only in Wordemann 2008 and Werff 2013; 6: Only in Werff 2013; 7: Only in Cooper 2017; 8: Only in Moncayo 2012. Included as potential confounder in Endara 2014, but discarded from the final multivariate logistic regression model; 9: Included as potential confounder in Moncayo 2012 and Endara 2014, but discarded from the final multivariate logistic regression model; 10: Only in Pereira 2007, no adjustment in Silva 2008; 11: Only in univariate analysis; 12: Other potential confounders may have been included in multivariate logistic regression model and discarded from the final model if did not alter OR by >10%, but not stated which; 13: only in 13-14 years old and rural residence; 14: Other variables with $p<0.25$ in univariate analysis may have been included in multivariate logistic regression model and discarded from the final model if $p>0.05$, but not stated which;. Han 2017

Supplementary Table 3: Results for the association between animal contact and wheeze/asthma

Study	Exposure		Outcome	Association	Measure	C.I. 95%	P value
**Brandao**	Living with cat or dog (if born in a public hospital)		Current wheeze	OR	1.03	0.60-1.76	0.893
	Living with cat or dog		Current wheeze	OR	0.99	0.77-1.27	0.982
**SCAALA Brazil**	**Rodents in home (vs. no)[1]**		**Non-atopic wheeze**	AOR	**1.68**	**1.21-2.34**	
			Atopic wheeze		0.79	0.54-1.15	
	**Cats in the house (vs. no)[1]**	Yes	Non-atopic wheeze	OR	1.35	0.79-2.29	
			Atopic wheeze		1.69	0.91-3.13	
		**Had in the past**	Non-atopic wheeze	OR	1.53	0.91-2.57	
			**Atopic wheeze**		**2.12**	**1.09-4.11**	
	Dog in the house (vs. no)[1]	Yes	Non-atopic wheeze	OR	1.11	0.76-1.63	
			Atopic wheeze		1.48	0.92-2.37	
		Had in the past	Non-atopic wheeze	OR	1.25	0.82-1.91	
			Atopic wheeze		1.39	0.86-2.26	
**Pelotas Study**	Pets at home (vs. No)[2]		Current asthma	OR	1.18		0.32
			Current wheeze	OR	1.21		0.25
**Cuban Study**	Current pet ownership (vs. no)		Current wheeze	OR	0.956		0.850
	Current pet contact (vs. no)				0.947		0.818
	Previous pet ownership (vs. no)				0.948		0.647
	Previous pet contact (vs. no)				1.229		0.155
	Pet inside house (vs.no)[3]				0.80	0.37-1.71	0.451
**ECUAVIDA**	Pet inside house (vs.no)		Current wheeze	AOR	1.01	0.74-1.37	0.974
			Asthma	AOR	1.26	0.82-1.93	0.300
	Large farm animals (vs. no)		Current wheeze	AOR	1.29	0.96-1.73	0.093
			Asthma	AOR	1.00	0.65-1.54	0.990
**SCAALA Ecuador**	Chicken outside house (vs. no)[4]		Current wheeze	AOR	1.24	0.86-1.77	0.244
	Cat inside house ever (vs. no)[4]		Current wheeze	AOR	1.26	1.00-1.59	0.051
			Atopic wheeze		1.45	0.85-2.46	0.631
			Non-atopic wheeze		1.25	0.94-1.66	
	Cat inside house presently (vs. no)		Atopic wheeze[4]	AOR	1.50	0.86-2.63	0.011
			Non-atopic wheeze[4]		0.76	0.56-1.02	
			Wheeze (rural)[5]	OR	0.74	0.48-1.15	0.183
			Wheeze (urban)[5]		1.05	0.62-1.80	0.853
	Pig around house presently (vs. no)[4]		Atopic wheeze	AOR	1.22	0.71-2.12	0.281
			Non-atopic wheeze		0.87	0.65-1.17	
	Pets inside the house (vs. no)[6]		Current wheeze	AOR	1.09	0.93-1.29	0.296
	**Dog inside house[5]**		Wheeze (rural)[5]	AOR	1.49	0.89-2.49	0.13
			**Wheeze (urban)[5]**		**2.37**	**1.09-5.12**	**0.03**
	Contact with farm animals (vs. no)		Atopic wheeze[4]	AOR	1.04	0.60-1.81	0.925
			Non-atopic wheeze[4]		1.01	0.74-1.39	
			Current wheeze[6]	AOR	1.03	0.85-1.25	0.781
			Wheeze (rural)[5]	OR	0.91	0.58-1.43	0.684
			Wheeze (urban)[5]		1.97	0.46-8.45	0.362
**Azalim 2014**	Presence of pets (vs. no)		Current asthma	OR	0.9	0.7-1.3	
**Barra. 2003**	Animals in home (vs. no)		Current wheeze	OR	1.12	0.95-1.38	
**Barreto 2013**	**Cat first year of life (vs. no)**		**Current wheeze**	**OR**	**0.2**	**0.1-0.7**	**<0.001**
**Casagrande 2008**	Dog in house first year life (vs. no)		Current wheeze	AOR	1.6	0.9-3.0	
	Cat in the house currently (vs. no)			OR	1.0	0.6-1.9	1.00
	Cat in house first year of life (vs. no)				0.8	0.4-1.8	0.69
	Dog in the house currently (vs. no)				1.2	0.8-1.8	0.41
	Animals in house first year life (vs. no)				1.2	0.8-1.8	0.34
	Animals in the house currently (vs. no)				1.2	0.8-1.7	0.30
**Del Río 2006**	**Pregnant mother in contact farm animals (vs. no)**		**Cumulative asthma boys 6-7y**	**OR**	**1.86**	**1.14-3.03**	**0.012**
			Cumulative asthma girls 6-7y		1.73	0.92-3.26	0.090
			Cum asthma boys 6-7y		1.80	0.96-3.37	0.069
	**Cat in the last 12 months (vs. no)**		**Cumulative asthma girls 13-14y**	**OR**	**1.32**	**1.04-1.67**	**0.025**
			**Current asthma girls 13-14y**		**1.37**	**1.04-1.81**	**0.025**
**Freitas 2012**	**Cat indoor currently (vs. no)**		**Current wheeze (rural)**	**AOR**	**3.4**	**1.0-10.9**	**0.041**
**Garcia 2008**	**Cat in home last 12 months (vs.no)**		**Current asthma 6-7 years**	**AOR**	**1.5**	**1.0-2.3**	**0.036**
	Dog in home first year of life (vs. no)				1.3	1.0-1.7	0.076

	Dog in home last 12 months (vs. no)		Cur. asthma 14-14 y			1.2	0.9-1.5	0.242
**Gomes 2015**	Dog/cat in home last 12 months (vs.no)		Current asthma		OR	1.02	0.70-1.49	0.89
**Guim. 2015**	Living with domestic animals		Asthma symptoms		PR	1,18	0.89-1.56	0.27
**Han 2017**	Cat in the house previous year (13-14 years old)		Current wheeze	Yes	N(%)	79 (36)		0.16
				No	N(%)	624 (41)		
			Current asthma	Yes	N(%)	24 (32)		0.15
				No	N(%)	668 (41)		
	Cat in the house first year of life (6-7 years old)		Current wheeze	Yes	N(%)	63 (28.5)		>0.05
				No	N(%)	307 (23.9)		
			Current asthma	Yes	N(%)	14 (24.6)		>0.05
				No	N(%)	356 (24.6)		
	Dog in the house previous year (13-14 years old)		Current wheeze	Yes	N(%)	193 (89)		0.33
				No	N(%)	1318 (87)		
			Current asthma	Yes	N(%)	64 (88)		0.85
				No	N(%)	1427 (87)		
	Dog in the house first year of life (6-7 years old)		Current wheeze	Yes	N(%)	147 (66.5)		>0.05
				No	N(%)	869 (68.1)		
			Current asthma	Yes	N(%)	38 (66.7)		>0.05
				No	N(%)	978 (67.9)		
	Current contact with farm animals (13-14 years old)		Current wheeze	Yes	N(%)	29 (13)		0.90
				No	N(%)	198 (13)		
			Current asthma	Yes	N(%)	13 (18)		0.25
				No	N(%)	213 (13)		
	Contact with farm animals first year of life	13-14 years old	Current wheeze	Yes	N(%)	25 (11)		0.99
				No	N(%)	175 (11)		
			Current asthma	Yes	N(%)	9 (12)		0.85
				No	N(%)	189 (12)		
		6-7 years old	Current wheeze	Yes	N(%)	65 (29.7)		>0.05
				No	N(%)	348 (27.4)		
			Current asthma	Yes	N(%)	15 (25.9)		>0.05
				No	N(%)	398 (27.8)		
**Kuschnir 2007**	**Cat in domicile (vs. no)**		**Asthma**		**AOR**	**1.32**	**1.04-1.69**	**0.025**
	Dog in domicile (vs. no)				PR	1.04	0.83-1.30	0.70
**Lima 2012**	**Exposure to dog or cat (vs.no)**		**Current wheeze**		**OR**	**1.26**	**1.01-1.59**	**0.040**
**Maia 2004**	**Exposure to pets (vs. no)**		**Current wheeze**		**AOR**	**1.27**	**1.03-1.56**	**0.023**
**Palvo 2008**	**Current exposure to pets (vs. no)**		**Current wheeze**		**AOR**	**1.83**	**1.45-2.32**	**<0.01**
**Ribei. 2002**	Presence of a pet at home (vs. no)		Current asthma		OR	0.54	0.71-3.57	
**Rojas 2001**	**Animals in the house (vs. no)**		**Cur. wheeze urban**		**OR**	**1.41**	**1.27-1.60**	
			**Cur. wheeze rural**		**OR**	**1.96**	**1.48-2.60**	
**Tintori 2013**	**Dog in the house**		Current wheeze		**AOR**	**2.64**	**1.14-6.07**	**0.023**
	Cat in the house				AOR	0.45	0.16-1.27	0.131
**Solis-Soto 2013**	Current dog contact (vs. no)		Current asthma (WQ)		AOR	1.31	0.91-1.90	
			Current asthma (VQ)		AOR	1.23	0.65-2.31	
	Current cat contact (vs. no)		Current asthma (WQ)		AOR	1.09	0.86-1.39	
			Current asthma (VQ)		AOR	1.43	0.96-2.13	
	Current contact farm animals (vs. no)		Current asthma (WQ)		AOR	1.19	0.94-4.51	
			Current asthma (VQ)		AOR	0.97	0.66-1.44	
**Boneberger 2011**	Regular contact with pets (vs. no)		**Asthma (GINA)**		AOR	0.70	0.41-1.04	
	**Regular contact farm animal (vs. no)**					**0.38**	**0.17-0.85**	
**Cadore 2017**	**Contact with cats (vs. no)**		**Asthma (GINA)**		**AOR**	**2.73**	**1.03-7.27**	**0.04**
	Contact with dogs (vs. no)				OR	1.19	0.61-2.31	0.616
**Coelho 2016**	Intradomiciliary dog		Asthma (>3 wheezing episodes last 12m)		AOR	1.51	0.97-2.34	0.070
	Intradomiciliary cat				OR	0.75	0.49-1.15	0.189
	Intradomiciliary bird				OR	1.23	0.1-1.85	0.332
	Any other intradomiciliary furry animal				OR	1.06	0.50-2.22	0.883
	Other intradomiciliary animals				OR	1.78	0.79-4.00	0.158
**Juca 2012**	**Animals in house currently (vs. no)**		Current wheeze		AOR	**1.8**	**1.2-2.9**	0.01
	Animals in house first year life (vs.no)					1.3	0.7-2.5	0.41
**Mora. 2001**	**Pet contact (vs.no)**		**Recurrent wheezing**		**OR**	**0.23**	**0.10-0.51**	**<0.05**
**Oliveira-Santos 2014**	Dog outside home nowadays (vs. no)		**Current wheeze**		OR	1.02	0.98-1.06	0.249
	**Dog outside home 1st year (vs. no)**				**AOR**	**0.93**	**0.88-0.98**	**0.018**

SCAALA: Social Changes, Asthma and Allergy in Latin America; GINA: Global Initiative for Asthma; C.I: confidence interval; OR: odds ratio; AOR: adjusted odds ratio; PR: Prevalence ratio. 1: Barreto 2010; 2: Cross-sectional study at 4-5 years of age (Chatkin 2003). Toledo 2011: No measure of association between animal exposure and wheeze/asthma; 3: Werff 2013; 4: Cross-sectional study (Moncayo 2010); 5: Endara 2014; 6: Cooper 2014. Associations with p<0.05 in bold characters.

Supplementary Table 4: Studies reporting on older siblings and wheeze/asthma

Study	Exposure		Outcome		Association	Measure	C.I. 95%	P value
**Cuban Study**	Presence of siblings (vs. no)		Current wheeze		OR	1.17		0.355
						1.16[1]	0.55-2.46	0.70
**ECUAVIDA**	Birth order (vs 1st)	2nd-4th	Current wheeze		AOR	1.15	0.78-1.69	0.487
		≥5th			AOR	0.92	0.52-1.64	0.779
	Birth order (vs 1st)	2nd-4th	Asthma		AOR	1.43	0.82-2.49	0.206
		≥5th			AOR	1.46	0.64-3.32	0.372
**Zepeda 2016**	Presence of siblings (vs. no)		Recurrent wheezing		N (%)	5 (63)		NS
			No recurrent wheezing		N (%)	8 (57)		NS
**SCAALA Ecuador**	**Birth order ≥5th vs ≤4th**		**Current wheeze[2]**		**AOR**	**0.75**	**0.61-0.91**	**0.005**
			Wheeze (rural)[3]		OR	0.65	0.41-1.03	0.065
			Wheeze (urban)[3]			1.45	0.73-2.90	0.293
**Uruguaiana Study[4]**	**≥2 siblings**		Wheeze		**AOR**	1.1	0.8-1.5	>0.05
			**Active asthma**			**0.5**	**0.3-0.9**	**≤0.05**
**Casagrande 2008**	No older siblings (vs.yes)		Current wheeze		OR	1.1	0.8-1.7	0.51
**Freitas 2012**	**≥2 older siblings**		**Current wheeze (urban)**		**AOR**	**2.6**	**1.2-5.7**	**0.015**
**Han 2017**	Number of older siblings	None	Current wheeze (yes / no)	13-14 years old	N (%)	73 (33) / 534 (35)		0.74
		1-2			N (%)	114 (52) / 758 (49)		
		≥3			N (%)	32 (15) / 245 (16)		
	Number of older siblings	None		6-7 years old	N (%)	86 (38.4) / 490 (37.9)		>0.05
		1-2			N (%)	101 (45.1) / 584 (45.2)		
		≥3			N (%)	37 (16.5) / 219 (16.9)		
	Number of older siblings	None	Current asthma (yes / no)	13-14 years old	N (%)	26 (35) / 572 (35)		0.85
		1-2			N (%)	38 (51) / 860 (50)		
		≥3			N (%)	10 (14) / 264 (16)		
	Number of older siblings	None		6-7 years old	N (%)	26 (44.8) / 550 (37.7)		>0.05
		1-2			N (%)	26 (44.8) / 659 (45.2)		
		≥3			N (%)	6 (10.3) / 250 (17.1)		
**Kuschnir 2007**	**Firstborn**		Asthma (questionnaire)		**AOR**	**1.34**	**1.07-1.68**	**0.011**
**Boneberger 2011**	≥1 older sibling (vs. none)		Asthma (GINA)		AOR	0.84	0.53-1.33	
**Coelho 2016**	Has older brothers		Asthma (>3 wheezing episodes last 12m)		OR	1.1	0.73-1.52	0.772
**Oliveira 2014**	**Any older siblings (vs. no)**		**Current wheeze**		**AOR**	**0.94**	**0.91-0.98**	**0.005**

AOR: adjusted odds ratio; C.I: confidence interval; GINA: Global Initiative for Asthma; NS: not statistically significant; OR: odds ratio; 1: Werff 2013; 2: Cooper 2014; 3: Endara 2014; 4: Pereira 2007. Associations with p<0.05 in bold characters.

Supplementary Table 5: Results for the association between day-care and wheeze/asthma

Study	Exposure		Outcome		Association	Measure	C.I. 95%	P value
**Brandao**	Attendance daycare at 24 months		Current wheeze		OR	0.69	0.35-1.34	0.277
**SCAALA Brazil[1]**	**Having attended day-care (vs. no)**		**Non-at wheeze**		AOR	**1.52**	**1.01-2.29**	
			Atopic wheeze			0.83	0.49-1.41	
**Cuban Study**	Pre-school day-care attendance		Current wheeze		OR	0.89	0.39-2.00	0.769
**SCAALA Ecuador**	Attending day care[2]	No	Current wheeze		AOR	Ref.		0.254
		<1 year old				0.80	0.55-1.18	
		≥ 1 year old				1.11	0.87-1.42	
	Attended day care (vs. no)[3]				OR	0.99	0.84-1.16	0.889
**Casagrande 2008**	No attendance nursery/kindergarden (vs.yes)		Current wheeze		OR	1.1	0.7-1.6	0.68
	Attended day-care when >1 year old (vs. ?)					1.0	0.6-1.6	0.89
**Han 2017**	Day care attendance in the first year of life		Current wheeze	Yes	N(%)			
				No	N(%)			
			Current asthma	Yes	N(%)			
				No	N(%)			
**Ribeiro 2002**	Full time at school (vs. part-time)		Asthma		OR	1.69	0.66-4.31	
**Bonebe. 2011**	**Day-care attendance (vs. no)**		**Asthma (GINA)**		**AOR**	**0.31**	**0.10-0.94**	
**Coelho 2016**	Attended daycare or nursery		Asthma (>3 wheezing episodes last 12m)		OR	1.39	0.85-2.28	0.190
	**Attended kindergarten**				**AOR**	**1.67**	**1.10-2.66**	0.024

AOR: adjusted odds ratio; C.I: confidence interval; GINA: Global Initiative for Asthma; OR: odds ratio; SCAALA: Social Changes, Asthma and Allergy in Latin America. 1: Barreto 2010; 2: Moncayo 2010, 3: Cooper 2014. Associations with p<0.05 in bold characters. Note: Cuban Study (Wördemann 2008) did not represent the adjusted results for day-care attendance, as there was no association (p<0.005).

Supplementary Table 6: Results for the association between early life infections and wheeze or asthma

Study	Exposure		Outcome	Association	Measure	C.I. 95%	P value
Brandao	**Pneumonia ever in life**	**Born public hosp**	**Current wheeze at 6 years**	AOR	**2.87**	**1.22-6.76**	**0.015**
		**Born private hosp**			**7.27**	**2.01-26.26**	**0.024**
	**Bronchiolitis first year of life**		**Current wheeze**	**AOR**	**1.59**	**1.01-2.53**	**0.04**
SCAALA Brazil[1]	**Acute respiratory infections during first year of follow-up (vs. no)**	**For >1-7 days**	**Non-at wheeze**	AOR	**1.54**	**1.01-2.36**	
			Atopic wheeze		1.54	0.92-2.59	
		**For ≥8 days**	**Non-at wheeze**		**4.87**	**2.26-9.76**	
			Atopic wheeze		2.27	0.97-5.38	
	Gastro-intestinal infections during first year of follow-up (vs. no)	For >1-7 days	Non-at wheeze	AOR	1.09	0.67-1.80	
			Atopic wheeze		1.02	0.58-1.81	
		For ≥8 days	Non-at wheeze		1.59	0.95-2.66	
			Atopic wheeze		1.29	0.70-2.40	
Pelotas Study[2]	**Acute respiratory infection at 6 and/or 12 months (vs.no)**		**Transient wheeze**	**OR**	**1.57**		**0.002**
			**Persistent wheeze**	**OR**	**1.81**		**0.004**
	Diarrhoea at 6 and/or 12 months (vs. no)		Transient wheeze	OR	0.92		0.7
			Persistent wheeze	OR	0.84		0.6
ECUAVIDA	**Pneumonia to 13 months[3]**		**Current wheeze**	**AOR**	**2.32**	**1.39-3.87**	**0.001**
			**Asthma**	**AOR**	**4.71**	**2.60-8.54**	**<0.001**
	**Acute respiratory infections first year of life (episodes)[4]**	**0**	**Atopic wheeze**	**N (%)**	**0 (0)**		**0.011**
			**Control**	**N (%)**	**2 (3)**		
		**1**	**Atopic wheeze**	**N (%)**	**3 (26)**		
			**Control**	**N (%)**	**35 (50)**		
		**2**	**Atopic wheeze**	**N (%)**	**11 (41)**		
			**Control**	**N (%)**	**27 (38)**		
		**3-5**	**Atopic wheeze**	**N (%)**	**9 (33)**		
			**Control**	**N (%)**	**6 (9)**		
	Acute diarrhea first year of life (vs. no)[4]		Atopic wheeze	N (%)	13 (48)		0.929
			Control	N (%)	33 (47)		
Zepeda 2016	RSV bronchiolitis (vs other viruses)		Recurrent wheezing	N (%)	5 (63)		NS
			No recur. wheezing	N (%)	8 (57)		
	RSV+RV bronchiolitis (vs other viruses)		Recurrent wheezing	N (%)	3 (27)		NS
			No recur. wheezing	N (%)	6 (43)		
Barr. 2003	**Resp. infection after birth (vs.no)**		**Current wheeze**	**AOR**	**3.44**	**2.76-4.69**	
Han 2017	**Bronchiolitis first year of life**		**Current wheeze**	**AOR**	**3.0**	**2.2-4.2**	**<0.01**
			**Current asthma**	**AOR**	**3.1**	**1.7-5.6**	**<0.01**
Pries. 2006	**Personal history of ARI**		**Recurrent wheezing**	**AOR**	**2.13**	**1.31-3.48**	0.002
Soto 1994	**>3 URTI / year (vs. 0-3)**		**Asthma**	**OR**	**4.25**		**<0.001**
Bone.2011	**Pneumonia first year of life (vs. no)**		**Asthma (GINA)**	**AOR**	**2.24**	**1.21-4.16**	

AOR: adjusted odds ratio; ARI: Acute respiratory infection; C.I: confidence interval; GINA: Global Initiative for Asthma; NS: not statistically significant; OR: odds ratio; SCAALA: Social Changes, Asthma and Allergy in Latin America; URTI: upper respiratory tract infections. 1: Barreto 2010; 2: Muiño 2008; 3: Cooper 2017; 4: Arrieta 2017. Associations with p<0.05 in bold characters.

Supplementary Table 7: Association between intestinal parasites and wheeze/asthma

Study	Exposure		Outcome	Association	Measure	C.I. 95%	P value
SCAALA Brazil	No *Ascaris lumbricoides* in stools (vs. yes)[1]		Current wheeze	AOR	0.72	0.51-1.02	
			Asthma ever		1.10	0.56-2.15	
	No *Trichuris trichiura* in stools (vs. yes)[1]		Current wheeze	AOR	0.92	0.61-1.41	
			Asthma ever		0.96	0.44-2.12	
	A. lumbricoides IgG4 positive (vs. negative)[2]		Non-atopic wheeze	AOR	1.25	0.79-1.97	
			Atopic wheeze		1.20	0.75-1.92	
	Toxocara canis IgG positive (vs. negative)[3]		Non-at wheeze + asthma	AOR	1.19	0.80-1.17	
			Atopic wheeze + asthma		1.16	0.74-1.82	
	Anti-Toxocara IgG levels (vs. negative: <0.23)[3]	0.23-0.99	Non-at wheeze + asthma	AOR	0.94	0.59-1.50	
			Atopic wheeze + asthma		1.15	0.69-1.94	
		≥1	Non-at wheeze + asthma	AOR	1.63	0.98-2.75	
			Atopic wheeze + asthma		1.20	0.66-2.17	
	Number of helminth infections[4]	1 vs 0	Asthma	AOR	1.02	0.78-1.34	
			Non-at asthma (vs non-at non-asthmatic		1.40	0.92-2.11	
			Atopic athma (vs non-atopic non-asthmatic		1.24	0.81-1.90	
			Atopic athma (vs atopic non-asthmatic		1.01	0.69-1.74	
		2 vs o	Asthma	AOR	1.20	0.82-1.75	
			Non-at asthma (vs non-at non-asthmatic		1.33	0.74-2.38	
			Atopic athma (vs non-atopic non-asthmatic		1.23	0.68-2.23	
			Atopic athma (vs atopic non-asthmatic		1.01	0.53-1.92	
		3 vs 0	Asthma	AOR	1.55	0.91-2.63	
			Non-at asthma (vs non-at non-asthmatic		1.84	0.87-3.89	
			Atopic athma (vs non-atopic non-asthmatic		0.91	0.36-2.34	
			Atopic athma (vs atopic non-asthmatic		1.02	0.37-2.84	
Cuban Study	**History of *A.lumbricoides*** (vs. no)[5]		**Current wheeze**	**OR**	**1.574**		**0.019**
	A. lumbricoides infection 3 years ago[6]		Current wheeze	OR	1.83	0.68-4.95	0.227
	T. Trichiura infection 3 years ago[6]		Current wheeze	OR	1.13	0.37-3.46	0.819
	History of hookworm (vs. no)[5]		Current wheeze	OR	1.541		0.287
	Hookworm infection 3 years ago[6]		Current wheeze	OR	0.74	0.24-2.31	0.62
	History of *E. vermicularis* (vs. no)[5]		Current wheeze	OR	1.35		0.092
	Current infect. any helminth (vs. no)[5]		Current wheeze	OR	1.012		0.932
	Current *A.lumbricoides* (vs. no)		Current wheeze	OR[5]	0.948		0.846
				AOR[7]	1.11	0.78-1.60	
			Asthma	AOR[7]	1.29	0.73-2.28	
	**Current hookworm (vs. no)**		**Current wheeze**	OR[5]	0.763		0.174
				**AOR[7]**	**0.43**	**0.27-0.69**	**<0.05**
			**Asthma**	**AOR[7]**	**0.53**	**0.32-0.87**	**<0.05**
	Current *E. vermicularis* (vs. no)[5]		Current wheeze	OR	0.902		0.795
	Current infection *T. trichiura* (vs. no)[5]		Current wheeze	OR	1.455		0.056
	**Current Toxocara (vs. no)[7]**		Current wheeze	AOR	1.13	0.69-1.84	
			**Asthma**	**AOR**	**1.51**	**1.01-2.26**	
ECUAVIDA	**Any maternal geohelminth**		**Current wheeze**	**AOR**	**1.41**	**1.06-1.88**	**0.017**
			**Asthma**	**AOR**	**1.28**	**0.85-1.94**	**0.238**
	**Any childhood geohelminth**		**Current wheeze**	**AOR**	**0.70**	**0.51-0.95**	**0.021**
			**Asthma**	**AOR**	**0.60**	**0.38-0.95**	**0.029**
	Any maternal geohelminth		Atopic current wheeze	AOR	0.75	0.38-1.47	0.396
			**Non-atopic current wheeze**	**AOR**	**1.60**	**1.16-2.20**	**0.005**
			Atopic asthma	AOR	1.68	0.64-4.41	0.292
			Non-atopic asthma	AOR	1.24	0.78-1.98	0.369
	Any childhood geohelminth (to 36 months)		Atopic current wheeze	AOR	1.38	0.70-2.71	0.351
			**Non-atopic current wheeze**	**AOR**	**0.60**	**0.43-0.86**	**0.005**
			Atopic asthma	AOR	1.27	0.49-3.28	0.620
			**Non-atopic asthma**	**AOR**	**0.52**	**0.31-0.87**	**0.014**

**SCAALA Ecuador**	**Intensity of *Trichuris trichiura***, (vs. negative)[8]	≤490 epg	Atopic wheeze	AOR	0.49	0.24-1.01	0.072
			Non-atopic wheeze		1.00	0.74-1.35	
		**>490 epg**	**Atopic wheeze**	**AOR**	**0.24**	**0.09-0.63**	0.010
			Non-atopic wheeze		1.00	0.70-1.43	
	Ascaris lumbricoides infection (vs. no)		Current wheeze	OR[9]	1.01	0.84-1.20	0.924
				AOR[10]	0.90	0.55-1.48	0.680
				AOR[8]	1.21	0.96-1.54	0.098
			Atopic wheeze[9]	AOR	0.76	0.37-1.55	0.447
			Non-atopic wheeze[9]	AOR	0.99	0.57-1.73	0.976
			Wheeze (rural)[11]	AOR	1.04	0.64-1.69	0.85
			Wheeze (urban)[11]		0.83	0.46-1.48	0.53
	***Trichuris trichiura* infection** (vs. no)		Current wheeze	OR[9]	1.16	0.98-1.36	0.084
				AOR[10]	0.72	0.44-1.18	0.187
			**Atopic wheeze**	AOR[10]	**0.47**	**0.22-0.98**	**0.043**
			Non-atopic wheeze	AOR[10]	0.82	0.46-1.47	0.510
			Wheeze (rural)[11]	AOR	1.09	0.58-2.03	0.783
			Wheeze (urban)[11]		1.70	0.78-3.69	0.177
	Any geohelminth (vs. no)		Current wheeze[9]	OR	1.12	0.95-1.34	0.165
			Wheeze (rural)[11]	AOR	1.21	0.68-2.18	0.52
			Wheeze (urban)[11]		1.22	0.56-2.66	0.62
	Hookworm (vs. no)[9]		Current wheeze	OR	1.10	0.77-1.58	0.591
**Uruguaiana Study**	Any helminths (vs. no)		Current wheeze[12]	OR	1.00	0.81-1.77	0.436
			Active asthma[12]		1.2	0.7-1.4	>0.05
			BHR[13]	OR	1.5	0.4-5.0	
	High load helminths[12]		Current wheeze	OR	1.6	0.7-1.9	>0.05
			Active asthma		1.8	1.0-2.7	>0.05
	**High-load helminth** (vs. <100 eggs/g)[13]		**BHR**	OR	**5.0**	**1.1-21.3**	
	**High load Ascaris[12]**		Current wheeze	**AOR**	1.8	0.9-3.6	>0.05
			Active asthma		2.4	0.98-3.4	>0.05
			**Non-at current wheeze**		**2.0**	**1.0-6.1**	**≤0.05**
			**Non-at active asthma**		**3.1**	**1.1-3.8**	**≤0.05**
	Giardia lamblia (vs. no)[13]		**BHR**	OR	**0.8**	**0.7-0.9**	
**Bragagnoli 2014**	*Ascaris lumbricoides*		Asthmatic (>3 wheezing episodes per year)	OR	1.00	0.53-1.90	0.99
	A. lumbric and *E. histolyitica/dispar*				0.50	0.23-1.09	0.08
	A. lumbric* and *T. trichiura				**2.31**	**1.20-4.45**	**0.011**
	A. lumbric, T. trich and *E. histol/dispar*				1.24	0.59-2.62	0.557
	A. lumb, E. histol/dispar and *G. lamblia*				0.61	0.20-1.90	0.396
	A. lumbricoides, T. trichiura , E. histol/dispar and *G. lamblia*				1.38	0.45-4.20	0.56
	A. lumbricoides and *G. lamblia*				0.47	0.10-2.21	0.336
	A. lumbric, T. trichiura and *G. lamblia*				0.37	0.04-3.13	0.35
	**Mild *A. lumbricoides* load**				**0.41**	**0.22-0.75**	**0.003**
	Intermediate *A. lumbricoides* load				0.89	0.38-2.09	0.79
	**Heavy *A. lumbricoides* load**				**2.37**	**1.35-4.18**	**0.002**
**Casag. 2008**	Worms (questionnaire) (vs. no)		Current wheeze	OR	1.2	0.7-2.2	0.54
**Cooper 2003**	Any geohelminth (vs. no)[14]		Current wheeze	AOR	0.82	0.51-1.32	0.4
	A. lumbricoides (vs. no)				0.88	0.58-1.34	0.5
	Trichuris trichiura (vs. no)				1.06	0.70-1.61	0.8
**Endara 2010**	Treatment with IVM (vs. no)[15]		Current wheeze	AOR	0.84	0.62-1.13	0.3
**Freitas 2012**	Helmintiasis		Current wheeze urban	OR	1.64		0.148
			Current wheeze rural		0.56		0.161
**Hagel 2007**	Prevalence of A.lumbricoides		BHR	RR	1.197	0.81-1.77	0.436
**Silva 2016**	Anti-Toxocara spp IgG seropositivity		Wheezing/asthma	AOR	1.14	0.69-1.90	
			Non-at wheeze (vs non-at non-wheeze)	OR	1.18	0.49-2.87	
			Atopic wheeze (vs. atopic non-wheeze)	OR	1.23	0.62-2.48	
**Cadore 2017**	Anti-Toxocara spp IgG seropositivity		Asthma (GINA)	AOR	1.89	0.52-6.89	0.34
**Coelho 2016**	Parasitological test (positive vs negative)		Asthma (>3 wheezing episodes last 12m)	OR	1.27	0.66-2.44	0.475
**Lopez 2009**	*Toxocara canis* infection (vs. no)		Asthma (Doctor's dx.)	OR	1.21	0.51-2.87	0.641
**Mendoza 2008**	**Intestinal parasitosis (vs.no)**		**Asthma** (GINA)	**PR**	**0.62**		**<0.05**
	A. lumbricoides (vs. no)				0.38		>0.05
	T. trichuria (vs. no)				0.53		>0.05
	Giardia lamblia (vs. no)				1.1		>0.05
	Entamoeba histolitica (vs. no)				0.7		>0.05
	Blastocystis hominis (vs. no)				0.63		>0.05
**Oliveir 2014**	Worm infection (question.) (vs. no)		Current wheeze	OR	0.97	0.92-1.01	0.210

SCAALA: Social Changes, Asthma and Allergy in Latin America; GINA: Global Initiative for Asthma; BHR: bronchial hyperresponsiveness; C.I: confidence interval; OR: odds ratio; AOR: adjusted odds ratio. PR: Prevalence ratio. 1: Alcantara 2012; 2: Barreto 2010; 3: Mendonça 2012; 4: Alcantara 2014; 5: Wördemann 2008; 6: Werff 2013; 7: Kanobana 2013; 8: Cross-sectional study (Moncayo 2010); 9: Cooper 2014; 10: Case-control study (Moncayo 2012); 11: Endara 2014; 12: Pereira 2007; 13: Silva 2008; 14: Adjusted for atopy; 15: No data for association between geohelminth infections and wheeze. Note: No association analysis in Moraes 2001 and Souza 2012 for the association between intestinal parasites and wheeze/asthma. Associations with $p<0.05$ in bold character.
